# Public health and the legal regulation of the pharmaceutical industry in Algeria

**DOI:** 10.11604/pamj.2022.41.86.31524

**Published:** 2022-02-01

**Authors:** Tareck Alsamara, Ghazi Farouk, Abbaci Adel

**Affiliations:** 1 Prince Sultan University, Riyadh, Kingdom of Saudi Arabia,; 2 Badji Mokhtar Annaba University, Annaba, Algeria

**Keywords:** Pharmaceutical industry, public health, Algeria, legal framework

## Abstract

Through this commentary, we attempt to evaluate the success of the Algerian legislature in developing a legal framework for the pharmaceutical industry that protects public health. The study is based on Algeria's national legal framework for the pharmaceutical industry, namely, legislation on health law and regulations on pharmaceutical activity. The study focuses on legal texts and uses the method of comparison whenever necessary. Algeria has established an integrated system for regulating the pharmaceutical industry at the national level. Algeria reserves its right to intervene in pharmaceutical economic activity through national industrial institutions. At the same time, Algeria allows private Algerian or foreign companies to become active in the pharmaceutical industry. However, the pharmaceutical industry sector has not freed the country from dependence on imports from abroad. Public authorities should provide continuous information about the support and facilities provided to pharmaceutical investors.

## Commentary

Algeria is a developing country, suffering from economic problems including economic dependence on the production of fossil fuels, and thus the pharmaceutical industry becomes an important alternative for the state to diversify its economy. In Algeria, sales of the drug reached 3.8 billion USD at the end of 2018 (Aissaoui 2020) [1]. In 2020 the amount was 4 billion USD and the forecast for 2021 is for a level of 5 billion USD (France business 2020) [2]. In 2021 the national production of drugs will have to exceed the value of 2.8 USD (APS 2021) [3]. The relationship between the pharmaceutical industry and public health is demonstrated by the fact that the pharmaceutical industry contributes to the achievement of public health goals. However, this relationship is not always beneficial, and Bloor and Maynard have questioned whether legislation on the pharmaceutical industry protects health or protects wealth [4].

The pharmaceutical industry has public health benefits but can become an enemy if the law does not define an exact relationship to protect society from scientifically harmful or unjustifiable medicines [5]. We must point out that pharmaceutical companies have contradictory obligations. This is because for they are obliged to make profit on the one hand, and to protect society from the rising costs of medicines on the other. Therefore, governments and non-governmental organizations play an important role in balancing these two contradictory commitments [6]. Moreover, the pharmaceutical industry has a positive impact on the availability of treatment and medicine, especially in developing and poor countries, but industrial companies tend to be more expensive. It is not always necessary to find a balance between social benefits and legal protection for companies. Pharmaceutical companies need to be protected. Intellectual property rights rules are not an obstacle to ensuring access of poor communities to essential medicines [7]. Therefore, international law tends to strike a balance between intellectual property rights and human rights norms relating to the right to health and access to medicines [8]. For example, the convention on the trade-related aspects of intellectual property rights imposes on countries the need to enact legislation to protect intellectual property, which many experts see as an obstacle to the availability of medicines in poor countries. But the 2001 Doha declaration to the convention's member states eased restrictions for the benefit of poor countries.

The pharmaceutical industry also contributes to ensuring the right to combat pain as a human right, because article 11 of the 1966 International Covenant on Economic, Social, and Cultural Rights affirms that health is a human right. The committee on economic, social, and cultural rights, the monitoring body of the convention, decided that states should provide and facilitate access to adequate quantities of public health, health care, goods, services, and programs [9]. Furthermore, the local pharmaceutical industries help countries to control epidemics and pandemics because they provide countries with medicines and medical supplies quickly and without the complications of importing. This makes it easier for countries to deal with health emergencies quickly and effectively. Algeria is aware of this, so Algeria is working to develop the local pharmaceutical industry. The COVID-19 pandemic has also had a profound impact on the medical and pharmaceutical industries. Because of it, governments now have to change their legislation to allow more effective treatments in health emergencies [10]. It has become necessary for governments to end foreign dependency in the pharmaceutical field by stimulating the national pharmaceutical economy. There have been no previous studies published in English about this subject. There is an article by Laouisset entitled “Algeria import constitution: the case of the pharmaceutical industry” [11], but it concerns import activity and is not related to the local industry. In Arabic, there is a study entitled “The role of industrial policies in the development of the pharmaceutical industry in Algeria”. The authors of the book, Ben Abdelrazzaq, and Khanshur, have a study that focuses on the economic aspects of Algerian national policy in the field of the medical industry but does not discuss the legal aspects.

The topic is particularly important because there is a direct relationship between the pharmaceutical industry and economic development [12]. Development of a national pharmaceutical industry not only helps to break free from dependence on imports. It also contributes to job creation [13]. Moreover, patents in the pharmaceutical industry contribute to the development of economic development and constitute an important source of wealth [14]. The need for a national pharmaceutical industry has also increased since the emergence of the COVID-19 pandemic, where the need for drugs to mitigate symptoms of the disease and for supplies to reduce the spread of the disease as well as its vaccine has increased [15]. Through this study, we are trying to evaluate the success of the Algerian legislature in developing a legal framework for the pharmaceutical industry that protects public health.

Executive Decree No. 92/284 of 6^th^ July 1992, regulated the procedure for registering medicines and pharmaceutical products used in human medicine and subjected them to strict procedures. Article 23 of the above-mentioned decree states that it is possible to refuse to register a drug if it is proven to be harmful or if its use does not achieve the expected therapeutic effect, or if it does not include a qualitative and qualitative structure or its contents, or if its production does not allow for quality control. We must point out that pharmaceutical activity is not exclusive to public enterprises but is also open to private companies, but that the practice of pharmaceutical products is subject to the licensing system 92/285 of 6^th^ July 1992, which contains the conditions to be met by the institutions involved in the production or distribution of pharmaceutical products. Because of the seriousness of public information and publicity relating to pharmaceutical products, the Algerian public authorities have established legal regulations for even this type of information and advertising, following executive Decree No. 92/286 of 6^th^ June 1992. According to the rules of this decree, pharmaceutical companies cannot make publicity without obtaining a visa from public authorities. The decree prohibits publicity for any prescription drug, or for treatments for specific diseases such as cancer, tuberculosis, AIDS, diabetes, infertility, blindness, or narcotic drugs.

The limitations of the commentary relate to the analytical method, which the study used in analyzing the legal texts related to the subject. We should note that the commentary produced the following findings: first, Algeria has established an integrated system for framing the pharmaceutical industry at the national level. Algeria reserves its right to intervene in pharmaceutical economic activity through national industrial institutions. At the same time, Algeria allows private Algerian or foreign companies to become active in the pharmaceutical industry. Second, Algeria has adopted institutions with considerable powers to regulate the pharmaceutical industry to maintain public health, the aim of which is to protect society. Third, under its legislative policy in the pharmaceutical industry, Algeria has struck a balance between the need to protect health on the one hand and the protection of wealth on the other. Fourth, Algeria still has several stages to invest in all legislative and institutional capacities in the pharmaceutical industry. Fifth, Algeria has a strong legal framework and institutions that allow it to invest in the pharmaceutical industry, especially during the COVID-19 pandemic. Sixth, Algeria responded positively to the situation in dealing with the COVID-19 pandemic and established the National Health Security Agency. It became the state adviser for strategic vigilance for future medical emergencies.

**The national laboratory for the control of pharmaceutical products:** the National Laboratory for the Control of Pharmaceutical Products is one of the oldest regulatory institutions in the pharmaceutical industry, established by decree 93/140 of 14^th^ June 1993. It is a public institution of an administrative nature with a legal character, whose functions are focused on monitoring the quality and quality of pharmaceuticals. It records medicines and studies the harmlessness, effectiveness, and quality of marketed medicines.

**The national pharmaceutical agency:** it is important to point out that the National Pharmaceutical Agency is a public institution with moral, personal and financial independence (article 2 of decree 1999/190). It's not a pharmaceutical company; it's a pharmaceutical industry control. The agency consists of a board of directors and a scientific board and is managed by a director. We note that issues relating to the agency's objectives are decided by the governing council following the legislation and organization in force. The governing council determines the agency's annual and multi-year projects, plans and program of work, and the agency's estimated budget. The agency's accounts, internal organization, and rules of procedure are also regulated. The council also examines draft transactions, contracts, agreements, and conventions, appoints a governor, and establishes regional attachments (article 10 of decree 1999/190). It should be noted that the board of directors of the agency consists of representatives of ministries of the state, such as the interior, as well as representatives of the ministries of health, labor, social security, and higher education. Three persons are appointed by the minister of health based on their competence and qualifications in areas related to the agency's functions (article 9 of decree 1999/190).

The scientific council of the agency differs from the board of directors. It is composed of representatives of the National Council for Health Ethics, the National Council for Medical Ethics, pharmaceutical workers, pharmacy organizations, patient associations, scientific and pharmaceutical associations, and university professors. It also contains five experts other than members of specialized committees appointed by the minister of health based on their competence and qualifications in areas relevant to the agency's functions (article 21 of decree 1999/190). It is necessary to note that the International Atomic Energy Agency (IAEA) scientific council is an advisory body, providing views and recommendations on matters relating to the agency's activity. It can make suggestions on strategies for developing the pharmaceutical sector, and can propose measures that would encourage production of pharmaceuticals and medical supplies. It might also express its opinion on all matters relating to the scientific and pharmaceutical fields relevant to the activity of the agency including draft legislative and regulatory texts governing pharmaceuticals and medical supplies (article 20 of Decree No. 1999/190). It must be made clear that the director of the agency is appointed by presidential decree on the recommendation of the minister in charge of health. Assisted in his duties by a secretary-general and sub-directors (article 16 of Decree 19/190), the director is responsible for implementing the decisions of the board of directors and the views of the scientific board of the agency. It is necessary to emphasize that the National Pharmaceutical Agency has exclusive competence in the area of registration, medicines, and pharmaceuticals. The agency is responsible for the registration, validation, and control of pharmaceuticals and medical supplies and participates in the implementation of the national policy on pharmaceuticals and medical supplies used in human medicine.

It is also responsible for the control of pharmaceuticals and medical supplies and is responsible for causing or requesting the competent authorities to take the necessary measures aimed at maintaining public health in the event of a pharmaceutical substance or medical requirement that constitutes or may pose a threat to human health (article 5 of Decree No. 19/190). To achieve its objectives, the agency may express an opinion on provisional licenses for the use of unregistered medicines. It also contributes to the definition of good practice rules for the manufacture, storage, distribution, and disbursement of pharmaceuticals. The agency's field inspection and inspection functions are carried out by inspectors including, in particular, the monitoring of the application of rules of pharmaceutical good practice and standards of medical supplies following the legislation and regulations in force. It also conducts scientific assessments of the benefits, risks, and therapeutic value of pharmaceuticals and medical supplies, as well as their economic aspects. It must be noted that its tasks include: the contribution to the preparation and updating of pharmaceutical codes and medical supplies, the contribution to the preparation of the list of pharmaceuticals and essential medical supplies, and the contribution to the preparation of the national pharmaceutical registry and the pharmaceutical constitution. The agency is also responsible for issuance of the drug price certificates upon registration as soon as it is determined by the joint sectoral commission on medicines, and the licensing the promotion and publicity of registered pharmaceuticals for health professionals. It gives opinions on the standards, rules of good practice, procedures, and methods applicable to medical studies in relation to pharmaceuticals and medical supplies. The agency undertake any study, research, or formative or information activities in the fields of its competence, and to contribute to the promotion of scientific research in the field of pharmaceuticals and medical supplies and the establishment of databases relating thereto. It participates in the preparation of the list of medicines compensable by social security bodies.

## Discussion

**The ministry of the pharmaceutical industry:** the most recent presidential Decree No. 20-163 of 23^rd^ June 2020 appointing members of the government as the ministry of the pharmaceutical industry, under the auspices of the ministry of health, has now been abandoned, but the scientific approach requires that its functions be recognized. Regarding the above-mentioned decree, it enjoyed extensive powers that overlapped with the functions of the National Agency for the Pharmaceutical Industry, such as: it prepare and promote pharmaceutical industry policy, national production, and policies for the promotion and development of investment in the pharmaceutical industry. It also develops and proposes measures and actions aimed at the abundance, quality, and availability of pharmaceuticals and medical supplies. We should note that it is body responsible for the accreditation of pharmaceutical enterprises in the production, import, export, exploitation, and distribution of pharmaceuticals and medical supplies, as well as medical promotion companies and service providers.

In the area of industrial policy, and in the promotion of national production and investment, it prepared the industrial policy of the pharmaceutical branch and assessed its impact, proposing necessary adjustments, in liaison with the parties concerned. It also promoted and harmonized the productive capacities of pharmaceutical enterprises to produce pharmaceuticals and medical supplies following specific objectives and national priorities. In addition, every measure was taken to achieve the objectives set by the pharmaceutical industry policy and to follow up the implementation of its development programs. It also encouraged the development of production inputs to create and promote the industrial fabric of users necessary for the integration of the pharmaceutical industry. It identified the mechanisms necessary to promote innovation and technological development in the pharmaceutical industry. All actions aimed at developing training and rehabilitation capacities in the professions of the sector could be proposed and implemented in liaison with the parties concerned. All measures for investment promotion could be proposed, and whatever contributed to improving the environment for the pharmaceutical industry.

It also ensured the regulation of investment projects towards the production of high-value-added essential pharmaceuticals. It facilitated the establishment of industrial pharmaceutical enterprises and the promotion of contracts and partnerships between the public sector and the national and foreign private sectors in the pharmaceutical industry, in particular through the development, monitoring, and evaluation of a partnership program for industrial public enterprises (article 3 of decree 2000/271). It should be noted that the ministry has powers in the area of the supply of pharmaceuticals and medical supplies. The ministry prepares the registration and certification policy and ensures its development and implementation, particularly in its targeting of high-value-added items from national production. The existing legislation and regulations on the quality, effectiveness, and security of pharmaceuticals and medical supplies are respected. In addition, all measures are taken to ensure the availability of pharmaceuticals and medical supplies, particularly in the area of market control.

Temporary licenses for the use of unregistered medicines are also received in accordance with the legislation and regulation in force. Such legislation ensures that import programs for pharmaceuticals and medical supplies are carried out in integration with national production. Any measure aimed at controlling the national production of pharmaceuticals and medical supplies is proposed through the national territory (article 4 of decree 2000/271). It is necessary to point out that the ministry assigned to the pharmaceutical industry plays an important role in the area of promotion of studies, research, and development under article 8 of Decree No. 20/271, by promoting research and development within producing pharmaceutical enterprises and by proposing all incentive measures for R & D activity in the pharmaceutical industry. It also promotes innovation in the pharmaceutical industry and ensures the promotion and development of clinic studies and the delivery of licenses.

The ministry also has several powers in the area of strategic vigilance, in accordance with article 7 of Decree No. 20/271, which ensures that it follows the development and direction of the national, regional, and international pharmaceutical markets and takes all measures to ensure their balance. It ensures that all technological security devices are developed in the field of pharmaceutical industry activities. It also works on the establishment of a data bank and the preparation of periodic and circumstantial reports on the evaluation of the pharmaceutical industry. It promotes any measure that facilitates and enables clients to access new technologies in the pharmaceutical industry. The list of pharmaceuticals and essential medical supplies, as well as the national pharmaceutical registry, the pharmaceutical constitution, and the national codes of pharmaceuticals and medical supplies, are also prepared and updated.

**The national agency for health security:** following the emergence of the COVID-19 pandemic, Algeria established the National Agency for Health Security by presidential Decree No. 20/158 of 13^th^ June 2020. This agency serves as an advisory body for strategic vigilance in the service of Algeria's public authority. It is both a private and a public institution with legal personality and financial independence. It is an institution for monitoring, consultation, and oversight of the national health security strategy and for ensuring its implementation. The agency's primary task is to prepare and implement the national health security strategy. The agency is responsible for coordinating national programs for the prevention and control of threats and risks of health crises. The agency is the scientific adviser to the public authorities in the area of health security and the reform of the national public health system. It is important to note that the agency has advisory, scientific guidance, and strategic vigilance bodies composed of scientific personalities, experts, and specialists recognized as competent in their fields.

**The role of private companies:** in Algeria, the pharmaceutical industry is not only confined to public institutions and agencies but to private institutions as well. Within this framework, the Algerian legislator, in article No. 184 of the law No. 08-13 relating to the protection and promotion of health, has exclusively designated the pharmaceutical public institutions and the accredited private ones as producers, importers, and exporters of the pharmaceutical products. In that context, the creation of a pharmaceutical company to produce medicines requires prior approval for completion and another approval for opening. These two approvals are exclusively delivered by the minister in charge of the pharmaceutical industry as stipulated in article No. 17 of the executive Decree No. 21-82 relating to the pharmaceutical institutions and their accreditation requirements.

Accordingly, many private actors like (Biopharm, Hydrapharm-Beker, Inpha-Médis-Biocare, LDM, Pharmalliance, etc.) play a significant role in the production, import, export, distribution of medicines in Algeria. However, the contribution of these private players remains insufficient compared to the needs of the local market and the facilities provided by the public authorities in terms of legislation and real estate. Carrying out their work, these private companies may sign agreements with foreign laboratories to create a common project in Algeria. For example, the private pharmaceutical company Biopharm signed an agreement worth 15 million USD with the Indian group Cipla in February 2015, to market and distributes medicines for respiratory diseases. Biopharm also signed in the same year another agreement with the Anglo-Swedish AstraZeneca laboratory of over 50 million USD for the construction of a pharmaceutical manufacturing plant in Algeria [16].

It is noted that the Algerian authorities in the last years are working to reduce the bill of the medicinal imports through encouraging private investments and pushing efforts towards the generic products. The aim of this is to reduce the dependency on foreign companies and to minimize the penetration of the Algerian pharmaceutical market by the world pharmaceutical companies.

## Conclusion

The aim of the study is to examine the capacity of Algerian law in the field of the pharmaceutical industry. Algeria has shown interest in the pharmaceutical industry by preparing legal grounds for the activity of public and private enterprises in the field. But the legal grounds are beset by economic and cognitive problems. In order to strengthen the role of the private pharmaceutical companies, it is highly recommended that public authorities should provide continuous information about the support and facilities provided to pharmaceutical investors. Public and private partnerships should be given priority within the pharmaceutical industry of the state. We should note that startup pharmaceutical companies should be encouraged and accompanied by the public authorities. Finally pharmaceutical incubators should also be given priority in order to contribute effectively to the development of pharmaceutical research.
